# Analysis of tRNA ^Cys^ processing under salt stress in *Bacillus subtilis* spore outgrowth using RNA sequencing data

**DOI:** 10.12688/f1000research.23780.1

**Published:** 2020-06-03

**Authors:** Iván Arvizu Hernández, José Luis Hernández Flores, Juan Caballero Pérez, Héctor Gutiérrez Sánchez, Miguel Ángel Ramos López, Sergio Romero Gómez, Andrés Cruz Hernández, Carlos Saldaña Gutierrez, Erika Álvarez Hidalgo, George H. Jones, Juan Campos Guillén

**Affiliations:** 1 Facultad de Química, Universidad Autónoma de Querétaro, Cerro de las Campanas S/N, Querétaro, Qro., 76010, Mexico; 2 Laboratorio de Bioseguridad y Análisis de Riesgo, Departamento de Ingeniería Genética, Centro de Investigación y de Estudios Avanzados del IPN, Irapuato, Guanajuato, 36824, Mexico; 3 Databiology Ltd, Databiology Ltd, Oxford, Oxford, OX4 4GA, UK; 4 Escuela de Agronomía, Universidad De La Salle Bajío, Campus Campestre, León, Guanajuato, 37150, Mexico; 5 Department of Biology, Emory University, Atlanta, Georgia, 30322, USA

**Keywords:** B. subtilis spore germination, outgrowth, salt stress, tRNACys processing, RNA-Seq data

## Abstract

**Background:** In spore-forming bacteria, the molecular mechanisms of accumulation of transfer RNA (tRNA) during sporulation must be a priority as tRNAs play an essential role in protein synthesis during spore germination and outgrowth. However, tRNA processing has not been extensively studied in these conditions, and knowledge of these mechanisms is important to understand long-term stress survival.

**Methods:**To gain further insight into tRNA processing during spore germination and outgrowth, the expression of the single copy tRNA ^Cys^ gene was analyzed in the presence and absence of 1.2 M NaCl in *Bacillus subtilis* using RNA-Seq data obtained from the Gene Expression Omnibus (GEO) database. The CLC Genomics work bench 12.0.2 (CLC Bio, Aarhus, Denmark, https://www.qiagenbioinformatics.com/) was used to analyze reads from the tRNA ^Cys^ gene.

**Results:**The results show that spores store different populations of tRNA ^Cys^-related molecules.  One such population, representing 60% of total tRNA ^Cys^, was composed of tRNA ^Cys^ fragments.  Half of these fragments (3´-tRF) possessed CC, CCA or incorrect additions at the 3´end. tRNA ^Cys^ with correct CCA addition at the 3´end represented 23% of total tRNA ^Cys^, while with CC addition represented 9% of the total and with incorrect addition represented 7%. While an accumulation of tRNA ^Cys^ precursors was induced by upregulation of the *rrnD* operon under the control of  σ ^A ^-dependent promoters under both conditions investigated, salt stress produced only a modest effect on tRNA ^Cys^ expression and the accumulation of tRNA ^Cys ^related species.

**Conclusions**:The results demonstrate that tRNA ^Cys^ molecules resident in spores undergo dynamic processing to produce functional molecules that may play an essential role during protein synthesis.

## Introduction

When unfavorable environmental conditions affect cell growth, spore-forming bacteria activate and coordinate a temporal molecular program to assemble a dormant spore, which sequesters essential biomolecules for subsequent conversion back into growing cells. *Bacillus subtilis* sporulation has proved to be an extremely valuable model system for the study of the principal biomolecules that are stored and shielded during spore dormancy. Proteome analysis has revealed several categories of proteins that are stored in dormant *B. subtilis* spores, including proteins involved in ribosome biogenesis, carbon metabolism, RNA processing, and protein synthesis. These protein groups play important roles in spore germination and outgrowth ( Swarge *et al.*, 2019). In addition, dormant spores store RNA, which suggests that these RNAs play specific roles during dormancy or germination and outgrowth or are used as ribonucleotide reservoirs for *de novo* RNA synthesis during spore germination. Some of the RNAs are transcripts involved in the spore formation process, such as those coding for membrane proteins, transporters, RNA processing enzymes, and proteins involved in protein synthesis, modification and degradation ( Bassi *et al.*, 2016; Keijser *et al.*, 2007; Nagler *et al.*, 2016; Segev *et al.*, 2012). Among these RNA categories, tRNA has been detected in significant amounts in dormant spores, and represents an essential reservoir ( Nagler *et al.*, 2016). One aspect of RNA metabolism that has not been well studied during spore germination and outgrowth is tRNA processing, and knowledge of those mechanisms will be essential to our understanding of long term stress survival by the spores and of the possible role of stored tRNAs in the generation of vegetative cells ( Deutscher, 2006; Li & Deutscher, 1996; Li & Deutscher, 2002; Mohanty & Kushner, 2000; Ow & Kushner, 2002; Raynal *et al.*, 1998; Reuven *et al.*, 1997).

In *B. subtilis*, tRNA ^Cys^ is encoded by a single copy gene and its study has significant relevance to aspects of processing and to the understanding of the global factors that affect cysteine metabolism. Thus, our previous studies have shown that tRNA ^Cys^ processing during exponential growth in *B. subtilis* involves the ribonucleases RNase Z, PNPase, RNase R and the 3’ end modifying enzyme, CCAase. The activities of these enzymes converge in ways that have not yet been completely elucidated to facilitate CCA addition and tRNA ^Cys^ repair or degradation ( Campos-Guillén *et al.*, 2010; Campos-Guillén *et al.*, 2019). To date, however, similar studies have not been performed on tRNA ^Cys^ metabolism during spore germination and outgrowth in *B. subtilis*.

A dormant spore is highly organized within a dehydrated spore core, which is enveloped by a dense inner membrane, a germ cell wall, a cortex and a spore coat ( Setlow, 2003). Each spore component is formed by molecules that play major roles in protecting the spore from a broad range of detrimental environmental conditions such as radiation, heat, desiccation and chemicals ( Setlow, 2006). Under specific conditions, environmental nutrients are recognized by the germinant receptors (GRs) and promote biochemical changes such as the release of monovalent cations and Ca ^2+^-dipicolinate, and the cortex is hydrolyzed and rehydrated. These molecular changes initiate the germination process ( Setlow, 2006). After this first step, a phase called outgrowth follows, in which metabolic activity and molecular organization (“ripening”) convert the germinated spore into a growing cell. During the outgrowth phase, at least 30% of *B. subtilis* genes are activated ( Horsburgh *et al.*, 2001; Keijser *et al.*, 2007).

One interesting aspect of spore germination is the effect of salt stress on the process and thus changes in the gene expression profile of outgrowing *B. subtilis* spores in the presence of high concentrations of NaCl have been analyzed by RNA sequencing ( Nagler & Moeller, 2015; Nagler *et al.*, 2014; Nagler *et al.*, 2015; Nagler *et al.*, 2016). In the last study, the presence of tRNAs in high percentage was detected in dormant spores but the nature of the tRNA population was not analyzed in depth. Taking advantage of new methodologies in high-throughput RNA sequencing, the availability of datasets in the Gene Expression Omnibus (GEO) database and computational analysis, we have investigated the profile of tRNA ^Cys^ in dormant and outgrowing *B. subtilis* spores in the presence of 1.2 M of NaCl. We were able to identify several categories of tRNA ^Cys^ species in dormant spores and during the first minutes of their conversion into growing cells. This study provides valuable insights into tRNA ^Cys^ processing during this state of transition.

## Methods

All experimental conditions to obtain RNA from dormant and outgrowing *B. subtilis* spores in the presence and absence of NaCl were previously described ( Nagler *et al.*, 2016). The raw RNA-Seq data indicated as SRR3488622 to SRR3488635 in Table 1, obtained from the Gene Expression Omnibus (GEO) database under the accession number GSE81238 were analyzed. The RNA-seq raw data correspond to: T0 (dormant spore), outgrowth of spores during 30 (T30), 60 (T60) and 90 (T90) minutes after the initiation of germination in the presence and absence of 1.2 M NaCl. Each sample time was sequenced twice.

To avoid contributions to our results by tRNA isoacceptors whose genes do not encode CCA at the 3´end in *B. subtilis*, we examined products of the tRNA ^Cys^ gene, which is present in single copy in the genome of *B. subtilis* and is located at the distal end of the *rrnD* operon. The gene does not encode the CCA at the 3´end, which must be added post-transcriptionally. The CLC Genomics work bench 12.0.2 (CLC Bio, Aarhus, Denmark) was used to analyze reads from the tRNA ^Cys^ gene, and small RNA adapters and reads with ambiguous nucleotides were trimmed using default settings. Open source alternatives such as UniPro UGENE, UTAP or Galaxy could also have been used for this purpose. Trimmed reads were then were mapped to the *B. subtilis* 168 genome downloaded from the NCBI website (accession number NC_000964.3) with short read local alignment mapping using the default setting. We considered alignments with a length fraction of 0.8 and a similarity fraction of 0.8. Two mismatches and three insertions and deletions per read were allowed. The mapped reads results for all experiments ranged from 243 to 1,141.

Mapped reads were used in the sequence alignment program MUSCLE in MEGA X v10.1.7 with default settings ( Kumar *et al.*, 2018) and high similarity aligned reads to the reference tRNA ^Cys^ sequence were manually counted and categorized as the following subpopulations: I) precursors (containing genomically encoded nucleotides at the 5´or 3´ ends), II) tRNA immature (these species were 71 nucleotides in length and lacked only the CCA-end), III) mature tRNA with 3´-CCA, IV) immature tRNA with 3´-CC. We also observed short, tRNA ^Cys^-derived RNA fragments (tRFs) in this study. Reads representing such fragments were categorized as V) 3´-tRF fragments detected with CCA 3’ends, VI) 3´-tRF fragments detected with CC 3’ends, VII) fragments detected as 5´-tRF (71 bases – *n*, where *n* > 0, from the 3’ end) or internal fragments (between nucleotide positions 2 to 70), VIII) complete tRNA ^Cys^ with incorrect 3´ tails and IX) 3´-tRF fragments with incorrect 3´ tails. The distribution analysis of tRNA ^Cys^ subpopulations was done using the JMP 7 program (SAS Institute Inc., Cary, NC) with the frequency per group algorithm. Trend graphs were constructed in SigmaPlot software version 14.0 (Systat Software, San Jose, CA).

**Table 1.** Distribution of mapped reads for tRNA ^Cys^ in dormant spore and outgrowth in presence and absence of 1.2 M NaCl.

GEO database	Experiment	Total reads	Mapped reads
**SRR3488627**	**T0**	**7,395,066**	**243**
**SRR3488623**	**T0**	**9,593,083**	**349**
**SRR3488631**	**No NaCl T30**	**11,077,704**	**414**
**SRR3488628**	**No NaCl T30**	**8,808,097**	**566**
**SRR3488632**	**No NaCl T60**	**10,264,883**	**785**
**SRR3488629**	**No NaCl T60**	**10,487,594**	**823**
**SRR3488633**	**No NaCl T90**	**12,877,261**	**1,141**
**SRR3488630**	**No NaCl T90**	**8,069,190**	**683**
**SRR3488624**	**NaCl T30**	**10,215,022**	**462**
**SRR3488622**	**NaCl T30**	**10,294,457**	**882**
**SRR3488634**	**NaCl T60**	**9,182,674**	**760**
**SRR3488625**	**NaCl T60**	**9,468,524**	**530**
**SRR3488635**	**NaCl T90**	**12,497,964**	**813**
**SRR3488626**	**NaCl T90**	**9,377,023**	**712**

GEO, Gene Expression Omnibus.

## Results

In order to provide in-depth insight into tRNA ^Cys^ processing in the dormant spore and during outgrowth in *B. subtilis*, RNA-Seq methodology and the GEO database ( GSE81238) were utilized. The total number of reads for all experiments ranged from 7 to 12 million, but an interesting finding was that there is a distribution of reads mapped for tRNA ^Cys^ in all experimental conditions tested ( Table 1).

### tRNA ^Cys^ mapped reads from dormant spores

Reads that mapped to the region of the tRNA ^Cys^ gene were used to distinguish between different stages of tRNA ^Cys^ processing. To reduce contributions to the analysis of modification-induced polymerase fall-off during RNA-Seq methodology and due to the high percentage of short tRNA ^Cys^-derived RNA fragments (5´-tRFs or 3´-tRFs) that were observed in the analysis, for purposes of statistical analysis, all mapped reads were characterized.

**Figure 1.**
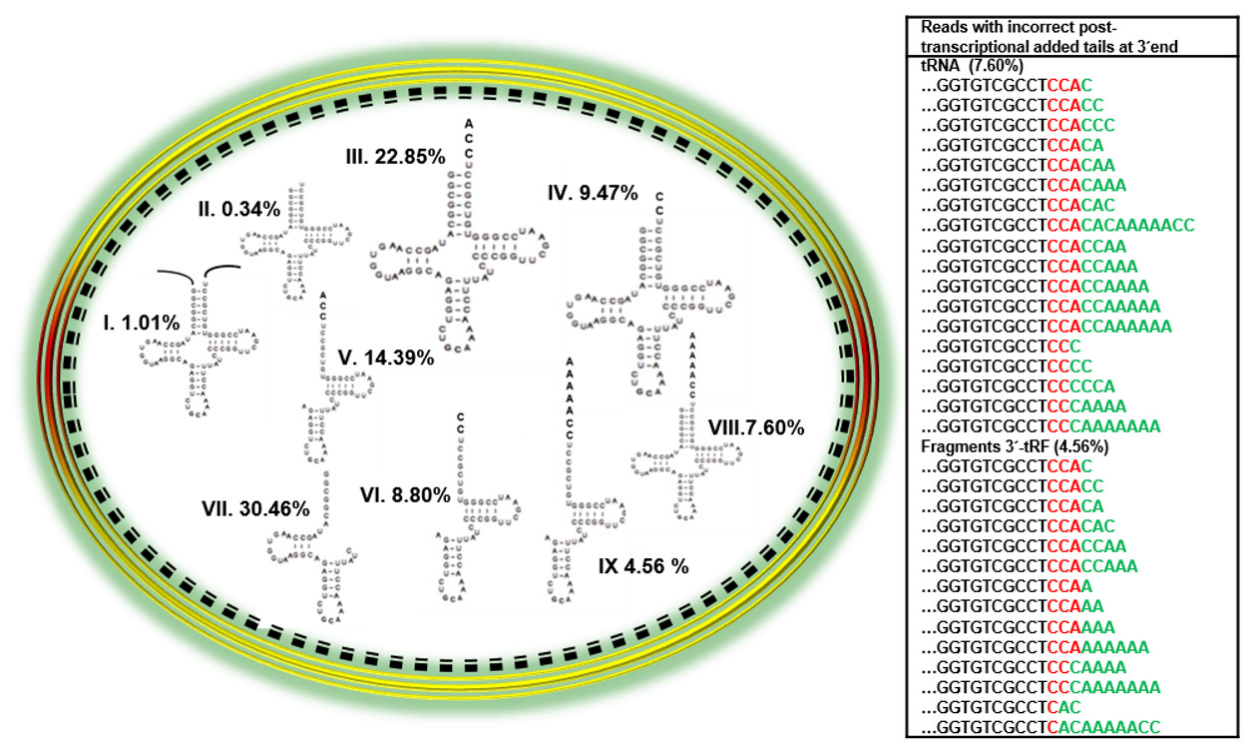
Overview of subpopulations of tRNA ^Cys^-related molecules in *B. subtilis* dormant spores. Reads categorized were: I) precursors, II) immature tRNA, III) mature tRNA with 3’ CCA ends, IV) immature tRNA with 3’ CC ends, V) 3’-tRF fragments detected with 3’ CCA ends, VI) 3’-tRF fragments detected with 3’ CC ends, VII) 5’-tRF or internal fragments, VIII) complete tRNA with incorrect 3´ tails added and IX) 3´-tRF fragments with incorrect 3´ tails added. The numbers after each category indicate the percentage of total tRNA ^Cys^ represented by that species. The table at the right shows the tails associated with the various tRNA ^Cys^ species in green.

The results show that the dormant spore stores a diverse population. An average of 296 mapped reads were analyzed in dormant spores (T0), and nine categories of tRNA ^Cys^-related species (see Methods) were characterized and are represented in Figure 1.

From these categories, we observed the following percentages of reads stored in dormant spores. A low percentage (1.01%) of reads represented tRNA with genomic encoded nucleotides at the 5´or 3´ ends, which were categorized as unprocessed transcripts (category I). The category with the lowest percentage of reads (0.34%) represented tRNAs with immature 3´ends (category II). The most abundant category of reads (22.85%) represented tRNAs with the correct CCA at the 3´ end (category III). An interesting finding was the relatively high percentage of mapped reads (9.47%) representing tRNAs with CC at the 3´end (category IV), which we suggest are immature tRNAs. We counted a high percentage of reads representing 3´-tRF fragments with correct CCA or CC 3´ends (23.19%) for categories V and VI. Due to the high number of mapped reads of fragments observed at time 0 (dormant spore) and compared with the lower number of mapped reads at the other times analyzed (see results below), we suggest that these reads are most likely fragments generated during spore formation. If these fragments are subsequently degraded, they might support *de novo* RNA synthesis during outgrowth. However, we cannot rule out the possibility that a percentage of them could be modification-induced polymerase fall-off byproducts resulting from the RNA-Seq methodology.

We observed a considerable percentage of reads (30.46%) of 5´-tRF or internal fragments, which are represented as category VII. The last categories observed, VIII and IX, were reads with incorrect post-transcriptionally added tails on complete tRNA ^Cys^ or 3´-tRF fragments. The total percentage of these reads was 12.16% and the tails are shown in Figure 1 (table at right side). Both categories of molecules of complete tRNA ^Cys^ or 3´-tRF fragments with CCA at the 3´end were observed to include species that carried the extra nucleotides C or CA. Some of them also had a second CCA terminus, while others possessed poly(A) tails.

### Distribution of tRNA ^Cys^ molecules during outgrowth

Our results show that the dormant spore stores mature tRNA ^Cys^, which could support the initial phases of translation, while tRNA ^Cys^ processing progress during the outgrowth phase. On the other hand, fragments might be degraded so that the ribonucleotides obtained could be available for *de novo* RNA synthesis during the outgrowth phase. Therefore, it is reasonable to suggest that during ripening phase, tRNA processing will progress and over time the population frequency of spore-stored tRNA ^Cys^ molecules will change.

**Figure 2.**
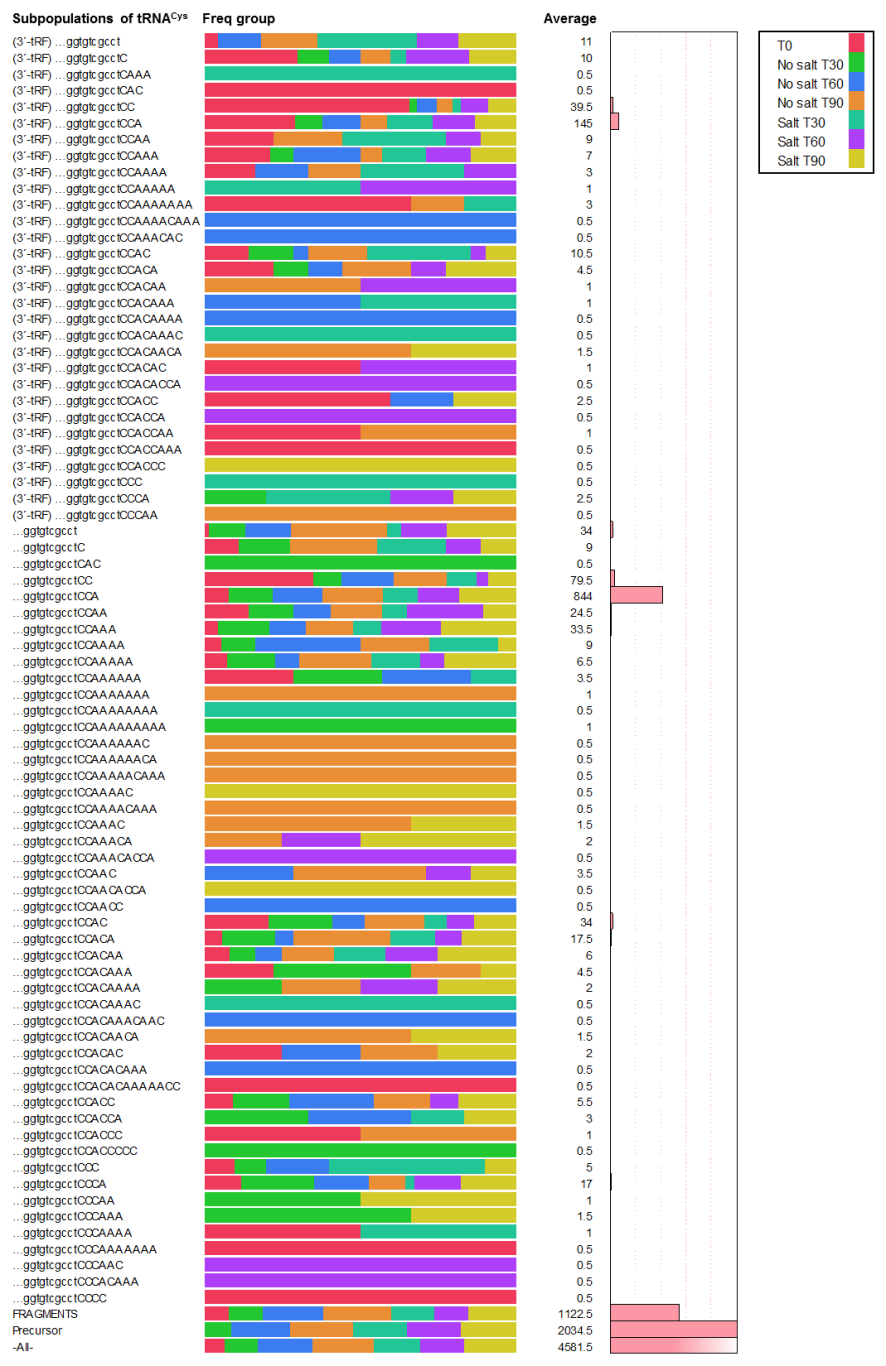
Distribution of subpopulations of tRNA ^Cys^ molecules in dormant spores (T0) and during the outgrowth process 30, 60 and 90 minutes after the initiation of germination in the absence and presence of 1.2 M of NaCl. We show the last ten nucleotides of tRNA ^Cys^, with correct or incorrect additions at the 3´end. Fragments (total 5´-tRF and internal fragments), 3´tRF and precursors are indicated at the left side. The average of mapped reads for each subpopulation is indicated by a number and by the bar graph at the right side and its presence at each sampled time by a colored bar in the graph.

To understand the population dynamics of tRNA ^Cys^ molecules during the outgrowth process, the mapped reads for tRNA ^Cys^ at 30, 60 and 90 minutes after the initiation of germination in the absence and presence of 1.2 M of NaCl were analyzed. From a total of 9,163 mapped reads obtained ( Table 1) during 90 minutes of outgrowth in both conditions, the distribution of each subpopulation of tRNA ^Cys^ molecules was obtained, represented as the average at each sample time and visualized in Figure 2.

While some subpopulations of these molecules were observed at lower frequency and detected in at least one RNA sample and at one sampling time, other sub-populations were present at all analyzed time points. In agreement with previous reports, robust transcription was induced during outgrowth. Thus, the sub-population of precursors of tRNA ^Cys^ are accumulated at all analyzed time points and represent a high frequency of tRNA molecules from the reads mapped with an average of 2034.5 mapped reads.

During ripening phase, it is reasonable to suppose that a high accumulation of the subpopulation of precursors during outgrowth will change the subpopulation of immature forms of tRNA ^Cys^ (category II). However, the analysis of mapped reads shows a lower frequency, with an average of 34 mapped reads. These results may indicate that tRNA ^Cys^ processing from precursors by endo or exo-ribonuclease activities progresses to some extent during the outgrowth phase to produce the immature forms of tRNA ^Cys^ which are then available for CCA addition. In fact, the mature form of tRNA ^Cys^ with CCA at its 3´end did increase in abundance during the outgrowth phase, with an average of 844 mapped reads, which means that CCA-adding activity is manifested during the ripening phase. Our results indicate that a significant fraction of the tRNA ^Cys^ subpopulations with no CCA-end, or with C- or CC-ends are converted to mature tRNA ^Cys^ during outgrowth. However, some immature species without complete CCA-ends were observed at all outgrowth time points.

An interesting subpopulation of tRNA ^Cys^ was observed and represented by fragments or 3´-tRFs with different additions at the 3´end; together, these subpopulations represent an average of 1365.5 mapped reads. These results indicate that during ripening phase, degradation by RNases is an important mechanism for obtaining ribonucleotides for RNA synthesis or that a mechanism of RNA quality control is operating in this phase. In this regard, an average of 194 mapped reads were observed for complete tRNA ^Cys^ molecules and an average of 57 mapped reads for 3´-tRF fragments with incorrect additions at 3´end.

### Distribution changes of tRNA ^Cys^ molecules during outgrowth at high salinity

**Figure 3.**
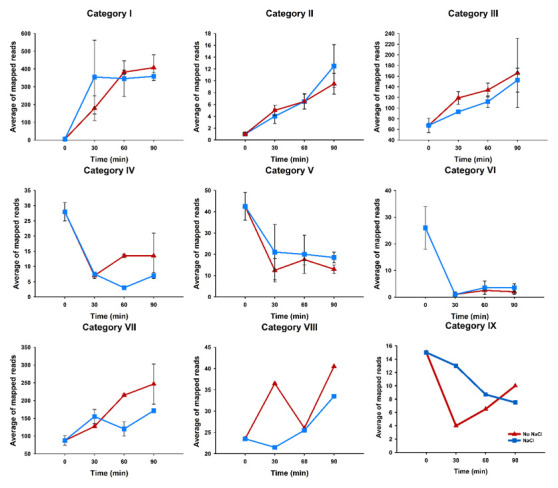
Overview of subpopulations of tRNA ^Cys^ molecules in *B. subtilis* dormant spores and during the outgrowth phase in the presence and absence of 1.2 M NaCl. Average of reads mapped for each category were plotted. Error lines represent standard errors of means.

Effects on gene expression under conditions of high salinity have been reported for outgrowing spores ( Nagler *et al.*, 2016). Therefore, to understand whether tRNA ^Cys^ processing and degradation might be affected by outgrowth in the presence of 1.2 M NaCl, mapped reads for each category mentioned above at the various sample times was contrasted with mapped reads from data obtained in the absence of NaCl. Our analysis results for mapped reads showed remarkably that precursors (category I) for tRNA ^Cys^ strongly increased at 30 minutes after the initiation of germination in both conditions investigated compared with reads from dormant spores (T0), no statistical difference was observed in the presence of NaCl. At 60 and 90 minutes, accumulation of precursors remains relatively stable and both conditions show similar averages of reads mapped ( Figure 3).

These results mean that during the ripening phase in outgrowing spores, tRNA ^Cys^ gene expression is upregulated. Given this observation, we investigated whether increased tRNA ^Cys^ transcription would differentially affect a particular subpopulation of tRNA ^Cys^ molecules related to the processing mechanisms. Beginning with category II, our results show that for the immature form of tRNA ^Cys^ there was an increase in abundance during outgrowth, but the frequency of reads mapped did not change significantly in the presence or absence of NaCl at any time point ( Figure 3). The next step was to investigate whether the subpopulation of tRNA ^Cys^ with CCA at the 3´end (category III) would be affected during salt stress. No statistically significant differences in the abundance of category III species were observed in the presence or absence of NaCl at any time point ( Figure 3).

An intriguing result was obtained in the analysis of immature tRNA with CC at the 3´end (category IV). While that subpopulation of tRNA ^Cys^ molecules decreased dramatically during the first 30 minutes after the initiation of germination in both conditions, in the presence of NaCl the abundance of this species continued to decrease, while in the absence of NaCl at 60 minutes this subpopulation of tRNA ^Cys^ molecules increased somewhat in abundance. These results suggest that CCA repair mechanisms may be operating in the absence of NaCl to produce mature tRNA ^Cys^ during this phase. If we consider the results for tRNA ^Cys^ stored in dormant spores at T0, categories III plus IV represent an average of 95.5±16 mapped reads, compared with an average of 93±1 and 119±12 mapped reads, respectively, for tRNA ^Cys^ with CCA in the presence and absence of NaCl at 30 minutes after the initiation of germination. These observations suggest that CCA-addition mechanisms are operative in the presence of NaCl, albeit less efficiently than in its absence. However, we cannot rule out the possibility that in the presence of NaCl RNase activity might be rerouted as a mechanism to obtain ribonucleotides to support transcription rather than CCA repair.

This last interpretation correlates with the results for the subpopulations of tRNA ^Cys^ 3´-tRF fragments with CCA or CC addition at the 3´end. The levels for both sub-populations (categories V and VI) decreased significantly, to near zero in the case of category VI, in both conditions during the outgrowth phase ( Figure 3). Figure 3 shows further that category VII fragments increased in abundance during outgrowth in the presence and absence of NaCl, but to a lower level in the presence of NaCl than that observed in its absence.

The abundance of category VIII species increased at T30 then decreased somewhat thereafter in the absence of NaCl, while a continuous increase in this species was observed during outgrowth in the presence of NaCl. At T90, category VIII levels were essentially the same in the presence and absence of NaCl. Category IX levels decreased during outgrowth, initially more dramatically in the absence of NaCl than in its presence.

## Discussion

In *B. subtilis,* tRNA genes are immersed in operons and tRNAs are therefore synthesized as precursors that undergo post-transcriptional modification to give functional molecules. These modifications include 5´and 3´ processing by ribonucleases, addition of CCA at the 3´end of those tRNAs that do not encode this sequence and post-transcriptional editing of ribonucleosides.

The tRNA ^Cys^ gene is present in single copy in the genome of *B. subtilis* and is located at the distal end of the *rrnD* operon, which codes for three rRNA molecules; 16S, 23S and 5S rRNA, and a cluster of tRNA genes found downstream of the 5S rRNA gene ( Vold, 1985; Wawrousek *et al.*, 1984). Regulation of expression of the *rrnD* operon is mediated by two cognate σ ^A^ consensus promoter sequences (P1 and P2) upstream of the 16S rRNA gene ( Koga *et al.*, 2006). A third σ ^A^ consensus promotor is localized in the 23S-5S intergenic space, and is associated with mechanisms of the stringent response ( Vold, 1985; Wawrousek *et al.*, 1984). A number of reports have revealed that the regulation of expression of P1 and P2 is maintained during spore formation, and that these promoters remain active even during the late stages of sporulation ( de Hoon *et al.*, 2010; Koga *et al.*, 2006; Rosenberg *et al.*, 2012). Other reports observed slight differences in the expression of the P1 and P2 promoters under nutritional stress in vegetative cells ( Natori *et al.*, 2009; Samarrai *et al.*, 2011).

Based on these reports, it is clear that tRNA ^Cys^ expression under the control of promoters P1 and P2 takes place during sporulation and that diverse sub-populations of tRNA ^Cys^ molecules might thus accumulate in dormant spores. To expand our knowledge of tRNA processing in dormant and germinating *B. subtilis* spores, RNA-Seq methodology was used to display the diversity of tRNA ^Cys^ species that were present in these conditions and in the spores germinated in the presence of high salt. Our results show for first time the diversity of the sub-populations of tRNA ^Cys^ stored in dormant spores in *B. subtilis* ( Figure 1) and contribute to the knowledge of tRNA ^Cys^ processing during the dormancy and outgrowth phases.

Our results show a progressive generation and degradation of tRNA ^Cys^ fragments ( Figure 3), which suggests that RNases are present and active at the various sampling times employed in these experiments. In fact, a recent report by Swarge *et al.* (2019) demonstrated via proteome analysis that several exo-ribonucleases are present in dormant spores, including PNPase, RNase R, RNase PH, YhaM, RNase Y and others. Transcripts for PNPase, YhaM and RNase Y were only observed at 15 minutes and for RNase R and RNAse PH at 30 minutes after the initiation of germination. Thus, these exo-ribonucleases remaining from sporulation are likely to play essential roles in the pathways of tRNA degradation and/or processing during the sporulation and ripening phases.

In the particular case of tRNA processing in *B. subtilis*, *in vitro* and *in vivo* studies of the endonucleolytic pathways of RNase P ( Evans *et al.*, 2006; Kurz *et al.*, 1998) or RNase Z ( Pellegrini *et al.*, 2003) and the exonucleolytic pathways of RNase PH, PNPase, RNase R or YhaM have been performed ( Oussenko *et al.*, 2002; Oussenko *et al.*, 2005). From these studies it is clear that tRNA precursors with extensions at the 5´ end are eliminated by RNase P, while RNase Z processes the 3´end of almost all tRNAs lacking an encoded CCA ( Pellegrini *et al.*, 2003) and the exonuclease RNase PH processes CCA-containing tRNA precursors ( Oussenko *et al.*, 2002; Oussenko *et al.*, 2005). The CCA motif at the 3´end is a key feature of all mature tRNAs and is essential for aminoacylation during protein synthesis ( Hou, 2010; Wellner *et al.*, 2018). Our results show that an important subpopulation of mature tRNA with CCA at its 3’ end is stored in the dormant spore and its levels increase during outgrowth. A delay in this increase was observed in the presence of NaCl, due perhaps to an effect of salt on the activities of enzymes related to tRNA ^Cys^ processing. In fact, there are reports demonstrating the effects on CCA addition to tRNA ^Cys^ of RNase Z, PNPase, RNase R and CCAase ( Campos-Guillén *et al.*, 2010; Campos Guillén *et al.*, 2019). It remains to be seen how these enzymes are affected under salt stress conditions during spore outgrowth, but Nagler *et al.* (2016) reported the interesting observation that the *cca* gene was downregulated during 90 minutes of outgrowth in the presence of NaCl. A relevant feature is that the *cca* gene is immersed in the operon containing bacillithiol (a REDOX regulator and an α-anomeric glycoside of L-cysteinyl-D-glucosamine with L-malic acid) biosynthetic genes under σ ^A^-dependent promoter control, which suggests that *cca* expression and the integrity of the 3’ terminus of tRNAs may be important during salt stress ( Campos Guillen *et al.*, 2017; Gaballa *et al.*, 2010; Gaballa *et al.*, 2013).

According to previous reports, incorrect additions at the 3´end of tRNA ^Cys^ were detected in *B. subtilis*, which suggests that alternative pathways of repair or degradation may exist in that organism ( Campos-Guillén *et al.*, 2010; Campos Guillén *et al.*, 2019; Cruz Hernández *et al*., 2013). Although a high percentage of mature tRNA with the CCA 3´end was observed in dormant spores, the species that would be available to support initial protein synthesis during outgrowth, it remains to be determined whether this stored tRNA ^Cys^ is charged with cysteine. It should be noted in this regard that cysteinyl-tRNA synthetase was detected in dormant spores and its expression was also detected at 60 minutes after the initiation of germination ( Nagler *et al.*, 2016; Swarge *et al.*, 2019).

Detrimental responses to salt during spore germination in *B. subtilis* have been analyzed previously ( Nagler & Moeller, 2015; Nagler *et al.*, 2014; Nagler *et al.*, 2015; Nagler *et al.*, 2016). From these studies, 402 genes related to the salt stress response were upregulated, some of them under σ ^A^-dependent promoter control ( Nagler *et al.*, 2016). Therefore, the observed accumulation of tRNA ^Cys^ precursors during the 90 minutes of outgrowth in our study suggests that upregulation of the *rrnD* operon, governed by σ ^A^-dependent promoters is important for macromolecular synthesis during outgrowth. An unanswered question is which of the three *rrnD* promoters is responsible for this upregulation.

## Conclusions

The ability to quantitatively monitor tRNA abundance changes from dormant spore to germination and outgrowth will be critical to understanding these processes and the roles of transcription, post-transcriptional modification and translation in them. The results reported here using RNA-Seq and data analysis provide new insights into the dynamic changes in the sub-populations of RNA species related to tRNA ^Cys^ molecules in dormant spores and during spore germination and outgrowth in the presence and absence of 1.2 M NaCl. An important unanswered question is what role these various subpopulations play in the metabolic economy of the spore and in the processes of germination and outgrowth.

## Data availability

### Source data

Raw RNA-Seq data on Gene Expression Omnibus, Accession number GSE81238: https://identifiers.org/geo:GSE81238

*B. subtilis* 168 genome from NCBI Reference Sequence, Accession number NC_000964.3: https://identifiers.org/refseq:NC_000964.3
