# Supplementary material for: Analysis of tRNA Cys processing under salt stress in Bacillus subtilis spore outgrowth using RNA sequencing data
Source: F1000Res. 2020 Jun 3;9:501. [Version 1] doi: 10.12688/f1000research.23780.1 (PMC8097732; doi:10.12688/f1000research.23780.1)
Supplement: Peer review history

# Peer review: Reviewer response for version 1

**DOI:** 10.5256/f1000research.26240.r83460

**Published:** 2021-05-10

**Authors:** José E. Barboza Corona

**Affiliations:** 1 Department of Food, Division of Life Sciences, Campus Irapuato-Salamanca, University of Guanajuato, Irapuato, Guanajuato, Mexico

This manuscript shows the different populations of tRNAcys-related molecules in dormant spores and during the first time of their conversion into growing cells in the presence or absence of saline stress. Different tRNA-cys species were categorized, and the percent of mature, immature or tRNA-cys fragments were determined.

 It is common that in nature, dormant *Bacillus* spores germinate in the presence of nutrients. In this sense, it would be fascinating in future works to determine if similar behavior is found under the presence of saline stress and nutrients. In this regard, I would like to see in the introduction some words explaining the foundation to use saline stress in this work and not the presence of nutrients to pass from dormant to germinated spores. As the primary condition to germinate spores was the presence of NaCl, it would be essential to explain briefly how this experiment was done (beginning of the methods).

 About the overview of the subpopulation of tRNA-cys in figure 3, I recommend using the annotation “No NaCl”, “NaCl” showed in the category IX figure, in all figures, or well, show this annotation in the figure 3 legend. Alternatively, one of my primary concerns of this figure 3 is that standard deviations are considerable in more of the times, so it is difficult to consider that statistically significant differences are not found in categories II and III in the presence or absence of sodium chloride as you indicated in the manuscript *. *You need to clarify this point.

Is the work clearly and accurately presented and does it cite the current literature?

Yes

If applicable, is the statistical analysis and its interpretation appropriate?

Partly

Are all the source data underlying the results available to ensure full reproducibility?

Yes

Is the study design appropriate and is the work technically sound?

Yes

Are the conclusions drawn adequately supported by the results?

Yes

Are sufficient details of methods and analysis provided to allow replication by others?

Partly

Reviewer Expertise:

Molecular studies with different Bacillus species.

I confirm that I have read this submission and believe that I have an appropriate level of expertise to confirm that it is of an acceptable scientific standard.

# Peer review: Reviewer response for version 1

**DOI:** 10.5256/f1000research.26240.r83556

**Published:** 2021-05-04

**Authors:** Fabian M. Commichau

**Affiliations:** 1 FG Synthetic Microbiology, Brandenburg University of Technology, Cottbus, Germany

In the present manuscript, Hernández and co-workers performed a bioinformatic analysis to uncover the modifications in the populations of tRNA ^Cys^-related molecules in the Gram-positive model bacterium *Bacillus subtilis*. The study is based on existing RNASeq data that have been generated with outgrowing spores in the absence and presence of sodium chloride. 

 The observation that the spores contain different tRNA ^Cys^ populations is very interesting. To the best of my knowledge, such an analysis has never been done before. 

 However, I have three critical points that I have to make:

 (i) the whole analysis is based on only two data sets. It is therefore not possible to carry out a statistical evaluation. This should be corrected in the text and in the figures.

 (ii) It is not entirely clear what functions the different tRNA populations could have in the cell. The authors could take up this point and speculate.

 (iii) Is it known that the addition of NaCl affects the maturation/modification of tRNAs? If so, what could be the underlying molecular mechanism?

Is the work clearly and accurately presented and does it cite the current literature?

Yes

If applicable, is the statistical analysis and its interpretation appropriate?

Partly

Are all the source data underlying the results available to ensure full reproducibility?

Partly

Is the study design appropriate and is the work technically sound?

Partly

Are the conclusions drawn adequately supported by the results?

Partly

Are sufficient details of methods and analysis provided to allow replication by others?

Yes

Reviewer Expertise:

I am not an expert in tRNA modification. My areas of expertise are bacterial physiology & genetics, gene regulation, second messengers.

I confirm that I have read this submission and believe that I have an appropriate level of expertise to confirm that it is of an acceptable scientific standard, however I have significant reservations, as outlined above.

# Peer review: Reviewer response for version 1

**DOI:** 10.5256/f1000research.26240.r82204

**Published:** 2021-04-19

**Authors:** Jungwook Kim

**Affiliations:** 1 Department of Chemistry, Gwangju Institute of Science and Technology, Gwangju, South Korea

By analyzing RNA-Seq data, the authors demonstrated dynamic population changes of diverse tRNA species from dormant to outgrowth phases of *Bacilus subtilis*. In the dormant phase, precursor RNA (Category I) is present only at an insignificant level, however, it becomes the most dominant species during the outgrowth phase. Precursor RNA apparently undergoes efficient maturation steps, where a relatively low level of immature species (Category II) was detected at all time points of the ripening stage. Interestingly, 5’-tRFs (designated as Category VII) are the second most abundant species next to precursor RNA during the outgrowth phase, showing a gradual increase in abundance over time. 3’-tRFs (Categories V, VI, VIII, and IX), on the other hand, appear to be formed at a much lower level in general compared to 5’-tRFs, and mature tRNA decrease sharply in the early stage of outgrowth. Authors proposed that these 3’-tRFs are intermediary species on the RNA degradation pathway during spore formation, products of which may serve as building blocks for de novo RNA synthesis in the outgrowth phase. The impact of a high concentration of salts (1.2M NaCl) on the distribution of tRNA subpopulation during the outgrowth phase was also investigated, but no significant effect was observed.

 The RNA-Seq analysis on dynamics of tRNA metabolism offers interesting biological insights into the regulation of tRNA processing in dormant and outgrowth phases of *B. subtilis*. Being a non-expert on RNA-Seq, however, interpretation of some data was not clear to me, for example, whether tRNA species with a small number of reads (one or less) are biologically relevant or merely represent sequencing artifacts. If the former, have those species been detected previously in other cases? Secondly, despite Category VII being the second most abundant species in both spore and ripening stages, the discussion on the biological significance of this class in *B. subtilis* was largely lacking. In particular, the size distribution of 5’-tRFs is not clearly described compared to that of 3’-tRFs. A representative structure of Category VII displayed in Figure 1 implies that the cleavage occurred around the variable loop of mature tRNA ^Cys^. Interestingly, previously known 5’-tRFs in eukaryotes include species much shorter in length, which are typically generated from the endonuclease activities on D- or stem-loops. Lastly, can tRNA or its fragment with CC instead of CCA at the 3’-terminus (Category IV and VI, respectively) be considered as a degradation intermediate derived from mature tRNA (Category III) as opposed to an intermediate resulting from incomplete addition of CCA from immature tRNA (Category II) as authors claimed?

Is the work clearly and accurately presented and does it cite the current literature?

Yes

If applicable, is the statistical analysis and its interpretation appropriate?

Partly

Are all the source data underlying the results available to ensure full reproducibility?

Yes

Is the study design appropriate and is the work technically sound?

Yes

Are the conclusions drawn adequately supported by the results?

Yes

Are sufficient details of methods and analysis provided to allow replication by others?

Yes

Reviewer Expertise:

tRNA modification

I confirm that I have read this submission and believe that I have an appropriate level of expertise to confirm that it is of an acceptable scientific standard.

# Peer review: Reviewer response for version 1

**DOI:** 10.5256/f1000research.26240.r77845

**Published:** 2021-02-05

**Authors:** Anne Moir

**Affiliations:** 1 Department of Molecular Biology and Biotechnology, University of Sheffield, Sheffield, UK

The authors present their *in silico* analysis of tRNA-Cys molecules in *B. subtilis* dormant spores, and during early outgrowth. The data analysed are those deposited by Nagler *et al.* (2016), in which they used RNA Seq to analyse gene expression during 90 min of outgrowth in an alanine glucose minimal medium. According to their published data, under these conditions the germinated spores do not yet show any increase in size, and this early stage of outgrowth has been referred to as “ripening”. The authors here have examined  all the data and have classified, for each relevant sequence read, the form of tRNA-Cys detected, from precursor through mature and degraded forms.  

 As the *in vivo* experiment was carried out in duplicate, with two samples at each time point, it is easy to overinterpret the absolute statistical significance of individual data points in Figure 3. I am not a statistics expert, but it seems that calculating a standard error of the mean of two values does highlight where the levels are particularly variable for an individual time point, but cannot be used in general to claim statistical significance.  Such qualitative experiments are extremely useful nonetheless, as data from the succession of time points confirm the general trend.  

 The major conclusions are (i) a variety of forms of tRNA-Cys are present in dormant spores; the relative proportions are described (though to a spurious degree of accuracy, as a result of too many significant figures used in the calculated percentages), (ii) that during outgrowth, the total levels of this tRNA species increase approximately three fold, and the relative amounts of the various forms change - most notably, the precursor form  increases dramatically, from almost negligible to approximately half of the total, and the mature CCA-tailed form increases approximately 2.5 fold in amount. There is a reduction in the level of several degradation products, except for that most commonly found (Class VII), the amount of which increases approximately three fold. There is no very strong evidence of differences between samples outgrowing in low and high salt in the graphs of Figure 3, although it may be the case for Category VII molecules.

 This is the first detailed analysis of tRNA-Cys levels in spores, and the implications for its synthesis and processing are fully discussed.

Is the work clearly and accurately presented and does it cite the current literature?

Yes

If applicable, is the statistical analysis and its interpretation appropriate?

Partly

Are all the source data underlying the results available to ensure full reproducibility?

Yes

Is the study design appropriate and is the work technically sound?

Yes

Are the conclusions drawn adequately supported by the results?

Partly

Are sufficient details of methods and analysis provided to allow replication by others?

Yes

Reviewer Expertise:

Spore germination and outgrowth in Bacillus subtilis.  I am not an expert in RNA Seq data or its analysis.

I confirm that I have read this submission and believe that I have an appropriate level of expertise to confirm that it is of an acceptable scientific standard, however I have significant reservations, as outlined above.

# Response

**Published:** 2021-02-17

**Authors:** JUAN CAMPOS GUILLEN

**Affiliations:** UNIVERSIDAD AUTONOMA DE QUERETARO, Mexico

Thank you for your comments. We agree with you about the interpretation of statistical significance in figure 3. However, we decided to use standard errors of means for the next reasons:  

 1. When the mapped reads for all categories for tRNA ^Cys^ were analyzed as percentage of total reads for each time, some data can easily be misunderstood. For example, for category III, from time 0 to time 30, a decrease percentage was observed when percentage of total was used. So that, the means for each category in each time was the better option for interpretation.

 2. The raw RNA-Seq data were obtained from Nagler et al., 2016, so that only duplicated data were obtained in these experiments. We were curious to analyze other Gene Expression Omnibus (GEO) database for *Bacillus subtilis*, using different RNA protocol and sequencing methods, however mapped reads for tRNA ^cys^ were not obtained.  This mean that as the only one copy gene for tRNA ^Cys^ in the genome, probably molar concentration could be less that other tRNAs isoacceptors with two or more copy genes. Additionally, RNA protocol and sequencing method could be affecting the total of mapped read, including in duplicated experiment.

 3. In view of the above and for a comprehensive solution to this issue, we use standard error as a mathematical tool used in statistics to measure variability. It is used to estimate the efficiency, accuracy, and consistency of a sample. In other words, it measures how precisely a sampling distribution represents a population. The standard error decrease as the sample size increases,  in our case, two values were used, but standard error of means show the distribution of mapped read from these samples at each time for each experiment for a correct interpretation in the presence and absence of 1.2 M NaCl. From these results, no statistical difference was observed in the presence of NaCl.
